# Genetically-Determined Polymorphism of Nonspecific Esterases and Phosphoglucomutase in Eight Introduced Breeds of the Silkworm, *Bombyx mori*, Raised in Bulgaria

**DOI:** 10.1673/031.008.1801

**Published:** 2008-03-11

**Authors:** Teodora Staykova

**Affiliations:** Department of Developmental Biology, section of Genetics, Faculty of Biology, University of Plovdiv, 4000 Plovdiv, Bulgaria

**Keywords:** isozymes

## Abstract

Isoenzymes are very suitable markers for the study of the inter-breed diversity of the silkworm *Bombyx mon* L. (Lepidoptera: Bombycidae). More than 250 breeds are raised in Bulgaria, which are not very well studied with regard to their isoenzymic polymorphism. Polymorphism of nonspecific esterases from pupal haemolymph was analyzed, as well as of phosphoglucomutase from different organs of larvae, pupae and imago, from eight introduced breeds. Electrophoresis in polyacrylamide gels was used. A polylocus control of nonspecific esterases, and possible monolocus control of phosphoglucomutase was ascertained. Biallele and triallele polymorphism of phosphoglucomutase locus and in three of the esterase loci was determined. The allelic frequencies of the polymorphic loci in each breed were analyzed. Inter-breed differences were found in different allelic frequencies, different heterozygosity and polymorphism.

## Introduction

**Figure 1.**
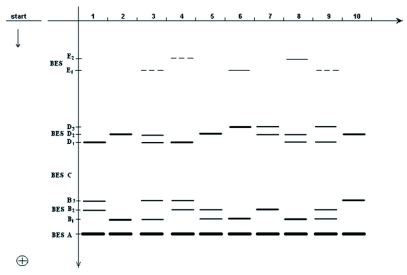
Esterase spectra *of Bombyx mori* haemolymph in 7.5% PAGE: 1–10, pupae 1–2 day.

The study of biodiversity in the silkworm is important for selection of useful traits. In order to find out the inter-breed variability of *Bombyx mori* L. (Lepidoptera: Bombycidae), enzyme markers have been found to be especially suitable (Patnaik and Datta 1995). With respect to their genetic structure enzymes are less changeable between individuals than other biochemical constituents of haemolymph, and other tissues (Etebari et al. 2005). This characteristic makes them good biochemical markers. Biodiversity among the more than 250 breeds of mulberry silkworm kept in Bulgaria (Petkov et al. 2006) has been studied mainly on the basis of the most important economic selection characters of quality and quantity. Isoenzymic polymorphism has been less studied in *B. mori* (Shabalina 1990; Stoykova et al. 2003), than genetically determined polymorphism in different enzymes (Eguchi et al. 1965; Hara et al. 1984; Egorova et al. 1985; Eguchi et al. 1988; Takeda et al. 1990; Takeda et al. 1993; Eguchi 1995; Sugimotto et al. 1995; Yamamoto et al. 2000). Within the breeds of *B. mori* raised in Bulgaria, the degree of variability of genetically determined isoenzymic polymorphism has not been previously studied. The objective of the present study was to examine the polymorphism of nonspecific esterases and phosphoglucomutase and on that basis to analyze the allelic frequency and degree of genetic heterogeneity with different breeds of mulberry silkworm introduced into Bulgaria from different ecological-geographic areas.

## Materials and methods

Individual samples from haemolymph, fat body, silk glands, gut, testes and ovaries of 480 female and male individuals taken from eight breeds of *B. mori* cultured in Bulgaria and known as Almaz, Asahi, Gindga 8, China 23, Kinshu, Maiak 5, Tokai and Showa. The breeds Almaz, Gindga 8 and Maiak 5 were introduced from Azerbaijan, the breeds Asahi, Kinshu, Tokai and Showa from Japan, and China 23 from China. Using 7.5% PAGE the spectrum of nonspecific esterases from different tissues and organs of 1–2 day old pupae were analyzed. Using 7.5% and 6% PAGE the spectrum of phosphoglucomutase of larvae, pupae and imago was studied. Polyacrylamide gel electrophoresis and isolation of haemolymph and organs were carried out according to Stoykova et al. (2003) and Staykova et al. (2004). Methods of Spencer et al. (1964) and Shaw and Prasad (1970) were used to visualize the phosphoglucomutase and the esterases respectively. Allele frequencies, heterozygosity (H) and polymorphism (P) were calculated after Ayala and Kiger (1987).

**Figure 2.**
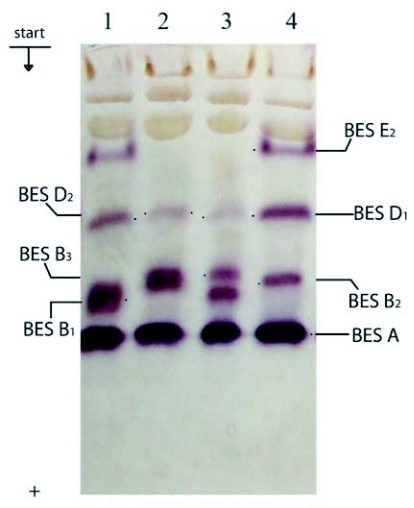
Esterase spectra of *Bombyx mori* haemolymph in 7.5% PAGE: 1–4, Maiak 5; pupae 1^st^ day.

## Results and Discussion

By means of comparative electrophoretic analysis of different tissues and organs of *B. mori*, it was found that for the study of inter-breed and intra-breed polymorphism hemolymph was most suitable for nonspecific esterases, and all tissues except the haemolymph were suitable for phosphoglucomutase.

**Figure 3.**
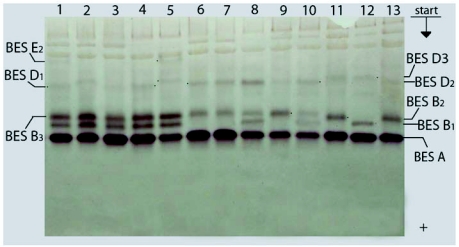
Esterase spectra of *Bombyx mori* haemolymph in 7.5% PAGE: 1–5, Kinshu; 6–10 - China 23; 11–13 -Giandga 8; pupae 1^st^ day.

### Nonspecific esterases

Nine esterase fractions belonging to four of the five previously described esterase zones (Stoykova et al. 2003) were found in haemolymph as shown in Figure 1 (BES A; BES B_1_, B_2_, B_3_; BES D_1_, D_2_, D_3_; BES E_1_ and E_2_). Esterases from zone BES C were not determined.

Breed specificity in the expression of the esterases from zones BES B, BES D and BES E was determined as follows:

**Figure 4.**
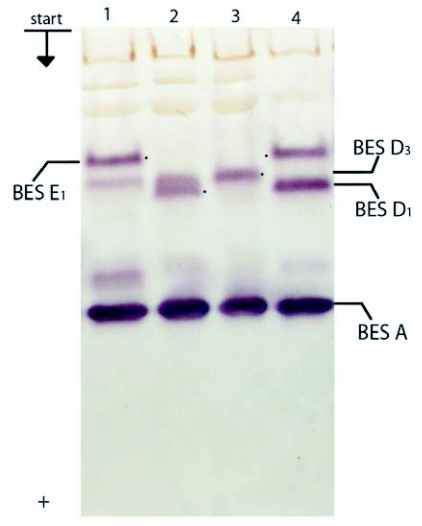
Esterase spectra of *Bombyx mori* haemolymph in 7.5% PAGE: 1–4, Giandga 8; pupae 2^nd^ day.

In zone BES B the bands BES B_1_, BES B_2_ and BES B_3_, were visualized each one separately or in pairs in the breeds Asahi, Gindga 8, China 23 and Maiak 5 (Figures 1, 2, 3). In the Tokai breed, only the bands BES B_1_ and BES B_2_ were present. In the Kinshu and Showa breeds, only BES B_1_ and BES B_3_ were present. In the haemolymph from the Almaz breed only fraction BES B_2_ was present.

In the Showa breed, only BES D_1_ was present. Fractions BES D_1_ and BES D_2_ were present separately or in combination in the breed Tokai, while BES D_2_ and BES D_3_ were present in the Asahi breed. Among the pupae studied from all the rest of breeds, fractions BES D_1_, BES D_2_ and BES D_3_ were present each one separately or in pairs (Figures 1, 2,3,4).

In zone BES E, in some individuals from Asahi breed, fractions BES E_1_ and BES E_2_ were present but showed different intensity (Figure 1). Fraction BES E_1_ was present in some individuals from the Almaz and Gindga 8 breeds (Figure 4), and BES E_2_, in some individuals from the Kinshu and Maiak 5 breeds (Figures 2, 3). In other individuals from these breeds, esterase bands from zone BES E were absent. With all studied individuals from China 23, Tokai and Showa, the absence of BES E esterase activity was also determined.

**Table 1.**
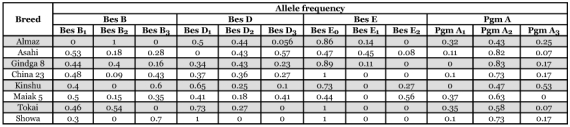
Allele frequencies at polymorphic esterase and phosphoglucomutase loci in the studied breeds of *Bombyx mori*.

The results obtained from this study confirmed the polymorphism established earlier for three of the esterase loci, marked as Bes B, Bes D and Bes E (Stoykova et al. 2003). The allele composition and the frequencies of different alleles was analyzed on the basis of this polymorphism. Breed specificity was ascertained (Table 1).

**Figure 5.**
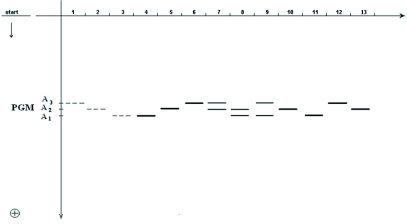
Phosphoglucomutase spectra of *Bombyx mori* tissues in 6% PAGE: 1, 2 and 3, haemolymph; 4–13,fat body, silk glands, testes and ovaries.

In the Asahi, Gindga 8, China 23 and Maiak 5 breeds, the three alleles in locus Bes B - Bes B_1_, Bes B_2_ and Bes B_3_ were present (Table 1). In the Tokai breed the alleles Bes B_1_ and Bes B_2_ were present, and in the Kinshu and Showa breeds, Bes B_1_ and Bes B_3_ were present. In the case of Almaz breed only allele Bes B_2_ was present. He (1995) and Stoykova et al. (2003) describe biallele polymorphism with codominant alleles on locus Bes B. The results obtained in this study when analyzing the breeds Kinshu, Tokai and Showa confirm these data. A third allele was found in the 5 remaining breeds, except Almaz.

He (1995) determined a triallele polymorphism of the esterases from zone BES D, and Egorova et al. (1985) found monomorphism in the same zone. The results obtained in this study suggest that triallele polymorphism in the locus Bes D with codominant alleles (Bes D_1_, Bes D_2_ and Bes D_3_) is present in the most analyzed breeds (Table 1). Only Showa is monomorphic in locus Bes D. In the Tokai breed, the alleles Bes D_1_ and Bes D_2_ were present, and in the Asahi Bes D_2_ and Bes D_3_ were present

The expression of the esterases from zone BES E, only in some individuals, and their absence in other individuals, as well as their different intensity of expression, suggests a triallele polymorphism in locus Bes E with a presence of a null allele (Bes E_0_, Bes E_1_ and Bes E_2_). These results confirm the earlier described polymorphism with null alleles in the same locus (Stoykova et al. 2003). Though, in some of the breeds analyzed in the present study, a third allele - Bes E2 was present. Among the studied individuals from the breeds China 23, Tokai and Showa only homozygotes in the null allele were present (Bes E_0_). In Almaz and Gindga 8 the allele Bes E_1_ was present, while in Kinshu and Maiak 5 the allele Bes E_2_ was present. In the case of the breed Asahi all three alleles were present (Table 1).

## Sex-dependent expression of haemolymph esterases was not established.

### Phosphoglucomutase

There was a lack of sex and stage specificity in the expression of the phosphoglucomutase of the various tissues and organs of larvae, pupae and adult of *B. mori*. Phosphoglucomutase is expressed in the fat body, the silk glands, the gut and the testes and ovaries. The expression of this enzyme in the haemolymph is low during all stages of ontogenesis (Figure 5).

**Figure 6.**
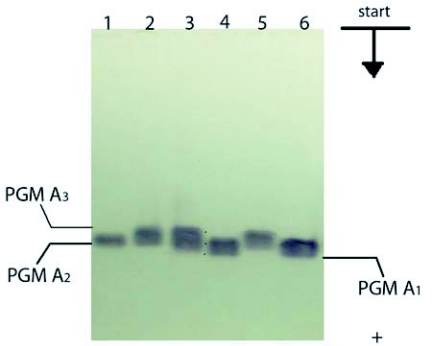
Phosphoglucomutase spectra of *Bombyx mori* in *6%* PAGE - silk glands of larvae 5th instar 7th day: 1–2, Gindga 8; 3–6, Almaz.

The comparative electrophoretic analysis of different specimens of the breeds studied showed the expression of three phosphoglucomutase fractions in all organs, labeled PGM A1, PGM A_2_ and PGM A_3_ in order of mobility (Figures 5, 6). These three bands were expressed independently or in pairs in the various individual spectra. The 6% polyacrilamide gel was found to be more appropriate for analysis of the polymorphism of phosphoglucomutase for *B. mori*.

The intensity and distribution of the three phosphoglucomutase bands in individuals suggest monolocus control with three codominant alleles, respectively Pgm A_1_, Pgm A_2_ and Pgm A_3_. Well-expressed inter-breed and intra-breed polymorphism was established in the Pgm A locus. The presence of the three alleles was established in Almaz, Asahi, China 23, Tokai and Showa (Table 1). The alleles Pgm A_1_ and Pgm A_2_ were present in Maiak 5, and Pgm A_2_ and Pgm A_3_ were present in Gindga 8 and Kinshu. There was breed specificity in differences in allelic composition, and differences in the frequencies of Pgm A alleles (Table 1). Interestingly, the allele Pgm A_2_ was present in all breeds studied, and in seven of these breeds this allele was present with the highest frequency. It was only in Kinshu that the frequency of the allele Pgm A_3_ was higher than the frequency of Pgm A_2_.

Phosphoglucomutase has not been previously used for a study on the biodiversity of *B. mori*. The polymorphism established provides evidence that this enzyme is a very suitable marker for the analysis of the inter-breed and intra-breed polymorphism.

**Table 2.**
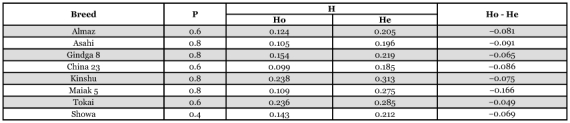
Polymorphism (P) and average heterozygosity (H) in the studied breeds of *Bombyx mori*: Ho - observed heterozygosity; He - expected heterozygosity.

### Heterozygosity and polymorphism

One of the indicators for the degree of genetic variability, which registers the average frequency of the heterozygote specimens in the population, is heterozygosity (H). The average heterozygosity (observed Ho and expected He) for every breed was established on the basis of the polymorphism determined and the allele and genotype frequencies calculated for each locus (Table 2). The heterozygosity observed had the highest value for Kinshu and the lowest value for China 23. The value of Ho was lowest in comparison with He for Maiak 5, and closest to the expected ones for Tokai. For all breeds studied the average value of Ho was lower than He, which is most likely due to the effects of inbreeding.

Another indicator of the level of genetic variability is its polymorphism (P). On the basis of the results obtained, the level of polymorphism for each of the breeds studied was calculated as well (Table 2). The highest value of P was found for Asahi, Gindga 8, Kinshu and Maiak 5, for which four polymorphic loci were described. The lowest value of P was found for the breed Showa.

### Conclusions

On the basis of the results obtained, the following conclusions can be made:

The nonspecific esterases of the haemolymph of *Bombyx mori* are under polygenic control and do not show sex specificity in expression.

Three of the esterase loci are polymorphic, and in two of the breeds studied additional alleles occurred, which has not been previously shown.

Phosphoglucomutase is expressed in all organs of *B. mori* and is possibly controlled by a polymorphic gene and does not show sex specificity.

Esterase and phosphoglucomutase are very suitable markers for analysis of the inter-breed and intra-breed polymorphism for the mulberry silkworm, and for determining the level of genetic variability.

There is a presence of breed specificity in allelic frequency and the heterozygosity of the breeds studied.

The polymorphisms shown for the nonspecific esterases and phosphoglucomutase, and their genetic variability can be expected to be present genetically different parents and could be used during selection to improve strains, (Konicheva et al. 1975; Egorova et al. 1985; Chatterjee et al. 1993; Patnaik & Datta 1995).
